# Potential Prospective Biomarkers for Non-small Cell Lung Cancer: Mini-Chromosome Maintenance Proteins

**DOI:** 10.3389/fgene.2021.587017

**Published:** 2021-04-14

**Authors:** Chen Huang, Chuqi Lei, Boyu Pan, Senbiao Fang, Yubao Chen, Wenfeng Cao, Liren Liu

**Affiliations:** ^1^Department of Gastrointestinal Cancer Biology, Tianjin Medical University Cancer Institute & Hospital, National Clinical Research Center for Cancer, Key Laboratory of Cancer Prevention and Therapy-Tianjin, Tianjin’s Clinical Research Center for Cancer, Tianjin, China; ^2^Hunan Provincial Key Lab on Bioinformatics, School of Computer Science and Engineering, Central South University, Changsha, China; ^3^Department of Computational Biology, Beijing Computing Center, Beijing, China; ^4^Department of Pathology, Tianjin Medical University Cancer Institute & Hospital, National Clinical Research Center for Cancer, Key Laboratory of Cancer Prevention and Therapy-Tianjin, Tianjin’s Clinical Research Center for Cancer, Tianjin, China

**Keywords:** MCM proteins, lung cancer, bioinformatics, prognosis, biomarkers

## Abstract

Minichromosome maintenance proteins (MCMs) are considered to be essential factors coupling DNA replication to both cell cycle progression and checkpoint regulation. Previous studies have shown that dysregulation of MCMs are implicated in tumorigenesis of lung cancer. However, the distinct expression/mutation patterns and prognostic values of MCMs in lung cancer have yet to be systematically elucidated. In the present study, we analyzed the transcriptional levels, mutations, and prognostic value of MCM1-10 in non-small cell lung cancer (NSCLC) patients using multiple bioinformatics tools, including ONCOMINE, GEPIA, Kaplan–Meier Plotter, cBioPortal, and GESA. The analysis results from GEPIA dataset showed that MCM2/4/10 was significantly high expressed in both lung adenocarcinoma (LUAD) and squamous cell lung carcinomas (LUSCs). Meanwhile, the expression levels of MCM2/4/6/7/8 were associated with advanced tumor stages. Subsequent survival analysis using the Kaplan–Meier Plotter indicated that high expression levels of MCM1/2/3/4/5/6/7/8/10 were associated with worse overall survival (OS), while high expression level of MCM9 predicted better OS in these patients. Furthermore, we experimentally validated overexpression of MCM2 and MCM4 in NSCLC, thus the results from this study support a view that they may serve as potential prospective biomarkers to identify high-risk subgroups of NSCLC patients.

## Introduction

Lung cancer is an common but highly fatal malignancy worldwide, with a 5-year overall survival (OS) varying from 4–17% depending on the tumor stage and regional differences (Lewis et al., 2010). Histologically, lung cancer can be classified into small cell lung cancer (SCLC) and non-small cell lung cancer (NSCLC). NSCLC accounts for 80% of total lung cancer cases, and can be further divided into lung adenocarcinoma (LUAD), squamous cell lung carcinoma (LUSC), and large cell lung carcinoma. The prognosis of NSCLC patients is poor, due to the late diagnosis and low sensitivity to traditional chemotherapy and radiotherapy, which raises an urgent need to identify novel biomarkers and potential therapeutic targets to improve the outcomes of NSCLC patients (Nielsen et al., 2012).

Minichromosome maintenance proteins (MCMs), as a group of proteins responsible for minichromosome maintenance, play essential roles in initiation of DNA replication and cell proliferation (Forsburg, 2004). Also, they are required for replication elongation, and implicated in cohesion, condensation, transcription, and recombination of DNA molecule (Forsburg, 2004). Evidence from yeast to human revealed that the six MCM proteins, namely MCM2-7, associated with each other to form a predominant heterohexameric structure, where each MCM member was present in equalstoichiometry in the cell (Bell and Stillman, 1992; Romanowski and Madine, 1996). Nevertheless, the other MCM subcomplexes with differing stoichiometries were also observed. For instance, a trimeric core complex of MCM4, MCM6, and MCM7 subunits could bind to MCM2, which in turn interacted with a peripheral dimer of MCM3 and MCM5 to form a hexameric MCM complex (Koonin, 1993). All MCM proteins are constitutively located in the nucleus throughout the entire cell cycle. Specifically, MCM2-7 binds to the origin part of replication at G1 phase, acting as the key regulatory components for DNA replication permission (Cannone et al., 2017).

Since MCM proteins are essential for initiation of DNA replication in dividing cells but not in quiescence cells, they are ideal markers for proliferation (Madine et al., 2000; Simon and Schwacha, 2014). Previous studies have shown that the expression level of MCMs is associated with the key clinicopathological parameters and exhibits significant diagnostic and prognostic value in multiple malignancies, including human oral, colon, ovarian, urothelial, and lung cancers (Giaginis et al., 2010; Gouji et al., 2011; Wang M. et al., 2019). In some cases, MCMs even exhibit higher specificity and sensitivity than the conventional proliferative markers, such as PCNA and Ki-67 (Madine et al., 2000). Meanwhile, several lines of evidence showed that MCMs could also serve as the markers for precancerous and recurrent conditions, and aberrant expressions of MCMs were implicated in the initiation and progression of many malignancies (Giaginis et al., 2010; Wu et al., 2018; Carlström et al., 2019). However, so far, the distinct expression/mutation patterns and prognostic values of MCMs in lung cancer have yet to be systematically elucidated.

In this study, utilizing multiple databases, we analyzed the expression and mutation status of MCMs in NSCLC, and evaluated the potential biological function as well as the prognostic values of MCMs in NSCLC patients, providing a bioinformatics-assisted strategy to facilitate the discovery of novel biomarkers or drug targets for lung cancer patients.

## Materials and Methods

### ONCOMINE Analysis

The ONCOMINE database^1^ was used as the cancer-related microarray data resource to analyze the transcription levels of MCMs in different cancers (Rhodes et al., 2004). The mRNA levels of MCM proteins in clinical cancer specimens were compared with that of normal controls, using a Students’ *t*-test to generate a *p* value. The cut-off of *p* value and fold change were defined as 0.01 and 2, respectively.

### Gene Expression Profiling Interactive Analysis Dataset

Gene Expression Profiling Interactive Analysis (GEPIA) is a recently developed interactive online server for analyzing the RNA sequencing data derived from the TCGA and GTEx projects through a standard processing pipeline. It can provide customizable functions, such as the tumor/normal differential expression analysis, the profiling according to cancer types or pathological stages, patient survival analysis, similar gene detection, correlation analysis, and dimensionality reduction analysis (Tang et al., 2017). In addition, GEPIA can further provide the transcripts per million (TPM) of the differential expression genes (DEGs) to show their relative expression levels.

### The Kaplan–Meier Plotter

The prognostic significance of the mRNA level of MCMs in lung cancer was evaluated using the Kaplan--Meier plotter^2^ (Győrffy et al., 2013), which contains gene expression data and survival information from 2,437 LC patients. To further analyze the OS of patients with lung cancer, the included patient samples were divided into two groups by median expression level (high *vs*. low expression level), and evaluated using Kaplan–Meier survival plot with the hazard ratio (HR) 95% confidence intervals (CI) and log rank *p* value. The *p*-value of less than 0.05 was considered statistically significant.

### TCGA Data and cBioPortal

Lung adenocarcinoma and LUSC datasets from The Cancer Genome Atlas (TCGA), including data from 503 and 466 cases with pathology reports, respectively, were selected for further analyses using cBioPortal^3^ (Cancer Genome Atlas Network, 2012). The analyzed genomic profiles included mutations, mRNA expression z-scores (RNA Seq V2 RSEM), protein expression Z-scores (RPPA) and putative copy-number alterations (CNA) from GISTIC. The network andco-expression was calculated following the online instruction of cBioPortal.

### Gene Set Enrichment Analysis

The gene expression profile of lung cancer was obtained from TCGA database^4^. The association between gene expression and biological processes was analyzed using Gene Set Enrichment Analysis (GSEA). The lung cancer samples were classified into the top and bottom 50% according to their expression levels (high vs. low levels). The default settings were used for the analysis and the thresholds for significance were determined by permutation analysis (1,000 permutations). The gene sets showing FDR of 0.25, a well-established cut-off for the identification of biologically relevant gene, were considered enriched between classes under comparison. The nominal *p*-value and normalized enrichment score (NES) were used to sort the pathways enriched in each phenotype. The KEGG gene sets were used for the enrichment analysis (Subramanian et al., 2005).

### Reagents

TRIzol reagent was obtained from Life technologies (United States), PrimeScript RT Master Mix and SYBR GREEN Premix reagents were obtained from TaKaRa (Japan).

### Patients and Tissues

Thirty fresh NSCLC tissues and paired-adjacent normal lung tissues were immediately frozen in liquid nitrogen after surgical removal in Tianjin Medical University Cancer Institute and Hospital (TMUCIH) from 2015 to 2016 and stored in liquid nitrogen until use. None of the NSCLC patients had been given chemo- and radiotherapy before surgery. Research protocols were approved by the Hospital Ethics Committee of TMUCIH, and the informed consents were obtained from all individual participants included in the study.

### RNA and Quantitative Real-Time PCR

Total RNA from NSCLC and paired normal lung tissues were extracted using the TRIzol reagent. RNA was quantified using Nano-drop 1000 (Thermo Fisher Scientific, United States). The 2 μg RNA from each sample was used for cDNA synthesis with PrimeScript RT Master Mix reagent according to the manufacturer’s protocol. By using the specific primer pairs (Supplementary Table 1) and SYBR GREEN Premix reagent, quantitative real-time PCR (qRT-PCR) was performed on the QuantStudio 5 real-time PCR system (Thermo Fisher Scientific, United States). Expression data were normalized to the geometric mean of the housekeeping GAPDH gene to control the variability in mRNA expression levels.

### Statistical Analysis

The qRT-PCR results were analyzed using SPSS17.0 software (United States). The differences expressed were using the Student’s *t*-test. *P* value of <0.05 was established to demonstrate significance in all statistical analyses.

## Results

### The Increased Transcriptional Levels of MCMs in Patients With Lung Cancer

**FIGURE 1:**
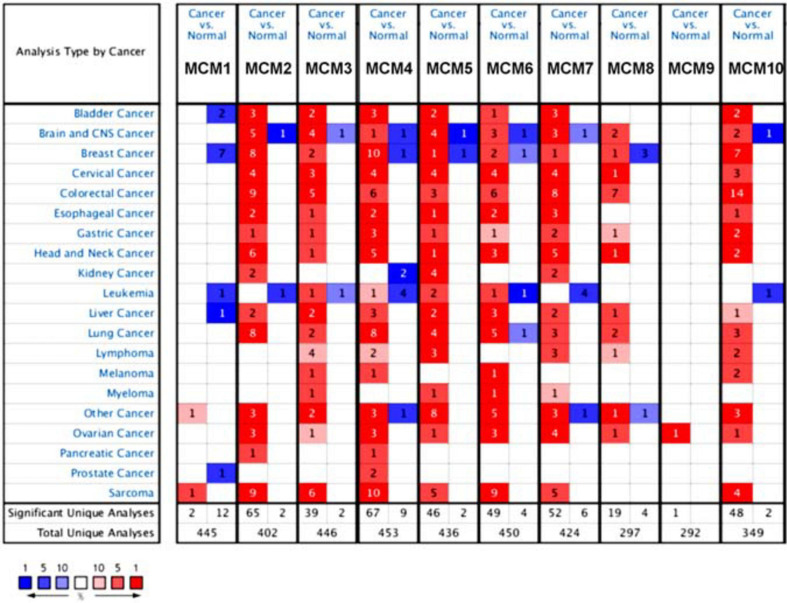
The transcription levels of MCMs factors in different types of cancers (ONCOMINE database). Red box represents a high expression in this cancer species, while the blue box indicates a low expression in this cancer species. The number in the box represents the number of relevant databases that meet the analysis results in the selected cancer species.

The transcription level of MCMs in cancer and normal samples was analyzed using the ONCOMINE database (Figure 1). The result showed that most of MCMs, except MCM1 and MCM9, were overexpressed in a wide range of malignancies, suggesting an overall correlation between the overexpression of MCMs and tumorigenesis. The same was true for lung cancer. Specifically, the mRNA level of MCM4 in patients with LUSC was significantly increased to a fold change of 14.79 in Bhattacharjee’s dataset. In Hou’s dataset, MCM4 was overexpressed in all of the NSCLC subtypes: in LUAD with a fold change of 3.39, in large-cell lung carcinoma with a fold change of 4.908, and in LUSC with a fold change of 5.794. In Garber’s dataset, patients with LUSC also showed overexpression of MCM4 with a fold change of 3.108 compared with the normal tissues. Similarly, increased mRNA level of MCM2 was observed in LUSC tissues in Wachi’s and Hou’s datasets with a fold change of 6.171 and 5.445, respectively. Meanwhile, analyzed with the same Hou’s database, the mRNA level of MCM2 was upregulated in large cell lung carcinoma and LUAD patients with a fold change of 5.129 and 3.25, respectively. These results thus showed higher upregulations of MCM4 and MCM2 in LUSC than those in LUAD and large cell carcinoma. Furthermore, MCM5 showed a high expression level with a fold change of 4.628 in LUSC samples in Bhattacharjee’s dataset, while MCM7 exhibiting an increased mRNA level in large-cell lung carcinoma with a fold change of 4.547 in Hou’s dataset. The mRNA level of MCM8 in LUSC (fold change = 3.587) and large-cell lung carcinoma (fold change = 3.919) were significantly increased than those in the normal samples in Hou’s dataset, and the mRNA level of MCM10 in LUSC and large-cell lung carcinoma were higher than those in normal lung tissues with the fold changes of 4.099 and 6.446, respectively (Table 1).

**TABLE 1:** The significant changes of MCMs expression in transcription level between different types of lung cancer and normal lung tissues (ONCOMINE database).

	**Types of lung cancer vs. lung**	**Fold change**	***P* value**	***t*-test**	**Data source**
SRF(MCM1)	NA	NA	NA	NA	NA
MCM2	Squamous cell lung carcinoma vs. normal	6.171	1.99E-05	12.132	Wachi
	Squamous cell lung carcinoma vs. normal	5.445	4.81E-22	18.676	Hou
	Lung adenocarcinoma vs. normal	3.251	3.46E-13	9.295	
	Large cell lung carcinoma vs. normal	5.129	6.10E-07	6.937	
	Squamous cell lung carcinoma vs. normal	2.204	1.13E-11	8.803	Talbot
	Lung adenocarcinoma vs. normal	2.436	3.20E-17	11.274	Selamat
	Lung adenocarcinoma vs. normal	2.411	2.36E-16	5.465	Su
	Lung adenocarcinoma vs. normal	2.12	3.44E-11	9.37	Okayama
MCM3	Squamous cell lung carcinoma vs. normal	2.387	8.85E-05	4.773	Garber
	Large cell lung carcinoma vs. normal	2.332	1.40E-05	5.421	Hou
MCM4	Squamous cell lung carcinoma vs. normal	5.794	2.02E-25	21.528	Hou
	Lung adenocarcinoma vs. normal	3.39	3.60E-15	10.484	
	Large cell lung carcinoma vs. normal	4.908	1.75E-08	8.895	
	Lung adenocarcinoma vs. normal	2.649	7.09E-10	8.123	Su
	Squamous cell lung carcinoma vs. normal	3.108	4.84E-07	8.298	Garber
	Lung adenocarcinoma vs. normal	2.403	8.50E-19	11.19	Landi
	Squamous cell lung carcinoma vs. normal	14.79	2.75E-06	5.355	Bhattacharjee
	Lung adenocarcinoma vs. normal	2.618	1.36E-18	11.673	Selamat
MCM5	Large cell lung carcinoma vs. normal	2.755	9.20E-05	8.632	Garber
	Squamous cell lung carcinoma vs. normal	2.889	2.51E-0.6	6.934	
	Squamous cell lung carcinoma vs. normal	4.628	7.91E-07	5.941	Bhattacharjee
	Squamous cell lung carcinoma vs. normal	2.305	7.88E-13	9.952	Hou
MCM6	Squamous cell lung carcinoma vs. normal	2.44	1.87E-11	8.071	Talbot
	Squamous cell lung carcinoma vs. normal	2.454	1.94E-05	5.906	Garber
	Squamous cell lung carcinoma vs. normal	2.65	6.45E-17	12.633	Hou
	Lung adenocarcinoma vs. normal	2.114	1.96E-12	8.473	
	Lung adenocarcinoma vs. normal	2.012	4.04E-06	5.67	Stearman
MCM7	Squamous cell lung carcinoma vs. normal	2.691	5.94E-15	13.573	Hou
	Large cell lung carcinoma vs. normal	4.547	4.11E-08	8.313	
	Lung adenocarcinoma vs. normal	2.336	1.06E-10	7.798	
MCM8	Large cell lung carcinoma vs. normal	3.919	1.03E-07	7.892	Hou
	Squamous cell lung carcinoma vs. normal	3.587	6.27E-12	10.719	
MCM9	NA	NA	NA	NA	NA
MCM10	Squamous cell lung carcinoma vs. normal	4.099	4.06E-16	14.598	Hou
	Large cell lung carcinoma vs. normal	6.446	7.96E-08	8.089	
	Lung adenocarcinoma vs. normal	2.894	1.28E-12	9.13	

**FIGURE 2:**
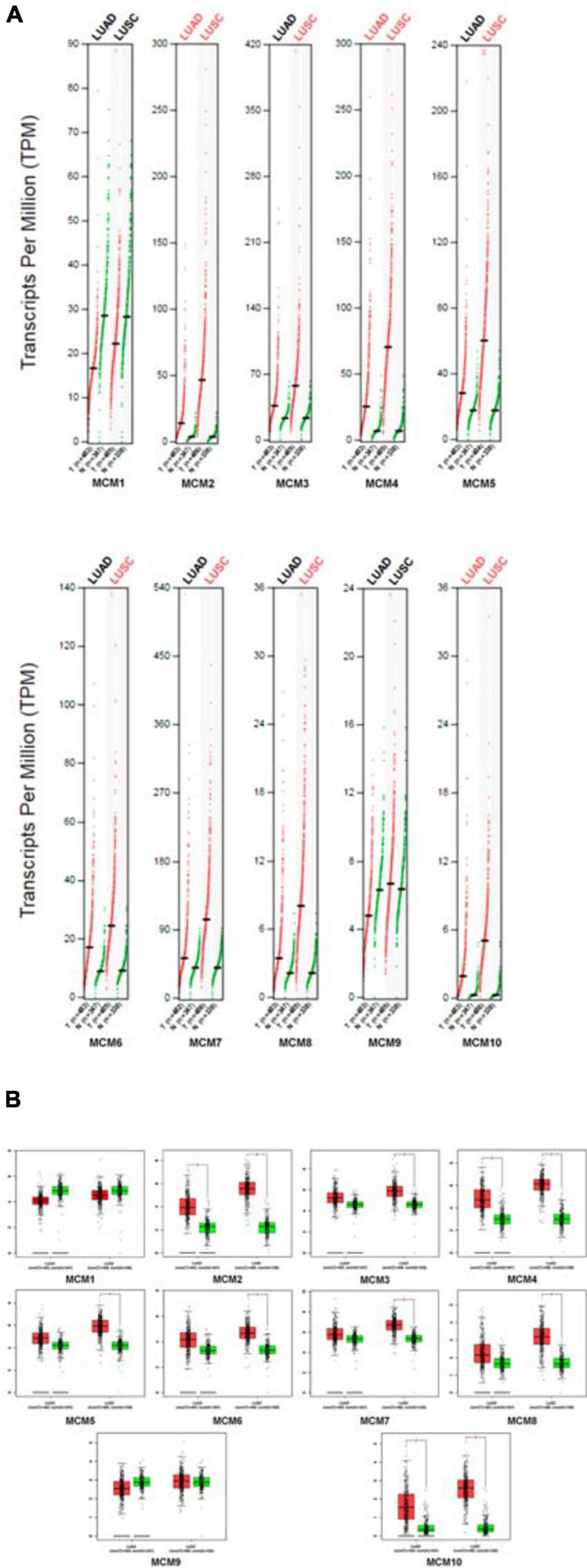
The expression levels of MCMs in NSCLC (including LUAD and LUSC) and normal tissue by GEPIA database. **(A)** Differential expression of MCMs between tumor and adjacent normal tissues from NSCLC described by TPM. The red dots represent the TPM value of each MCM in LUAD or LUSC tissues, while the green dots represent the TPM value of each MCM in the paired-normal tissues. T: Tumor, N: Normal. **(B)** Differences of expression levels between NSCLC and normal tissues described by box plots provided by GEPIA.

Next, we further determined the individual mRNA level of MCMs in NSCLC and that in normal lung tissues by using GEPIA online tools^5^. The result confirmed that the mRNA level of most MCMs, especially MCM2/4/10 were significantly higher in NSCLC tissues (LUAD or LUSC) than that in normal lung tissues (Figures 2A,B).

### The Expression Level of MCMs Was Correlated With Clinical Stages and Overall Survival in NSCLC Patients

**FIGURE 3:**
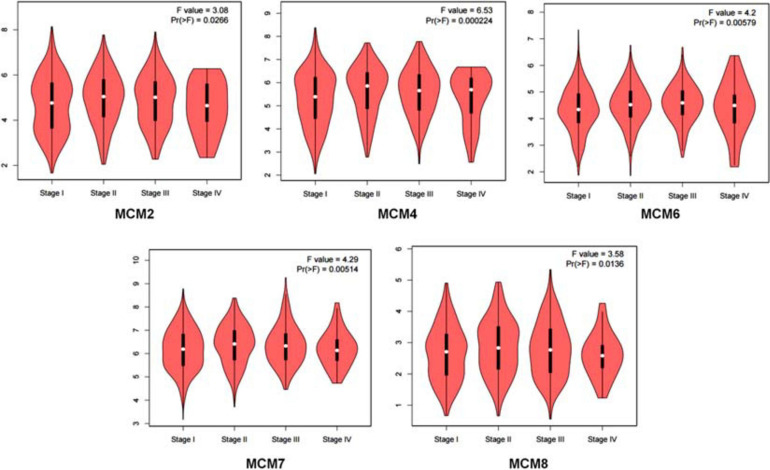
Correlation between MCMs’ mRNA level and clinical stages in lung cancer patients by GEPIA database. Describe by violin plots.

Having determined an overall aberrant expression of MCMs in NSCLC, we then analyzed the correlation between the mRNA level of MCMs and the clinical stages of LUAD and LUSC. The results showed that there was an varied but significant positive correlations between the expression level of MCM2/4/6/7/8 and the clinical stage of LUAD and LUSC patients [Pr (> F) < 0.03] (Figure 3).

**FIGURE 4:**
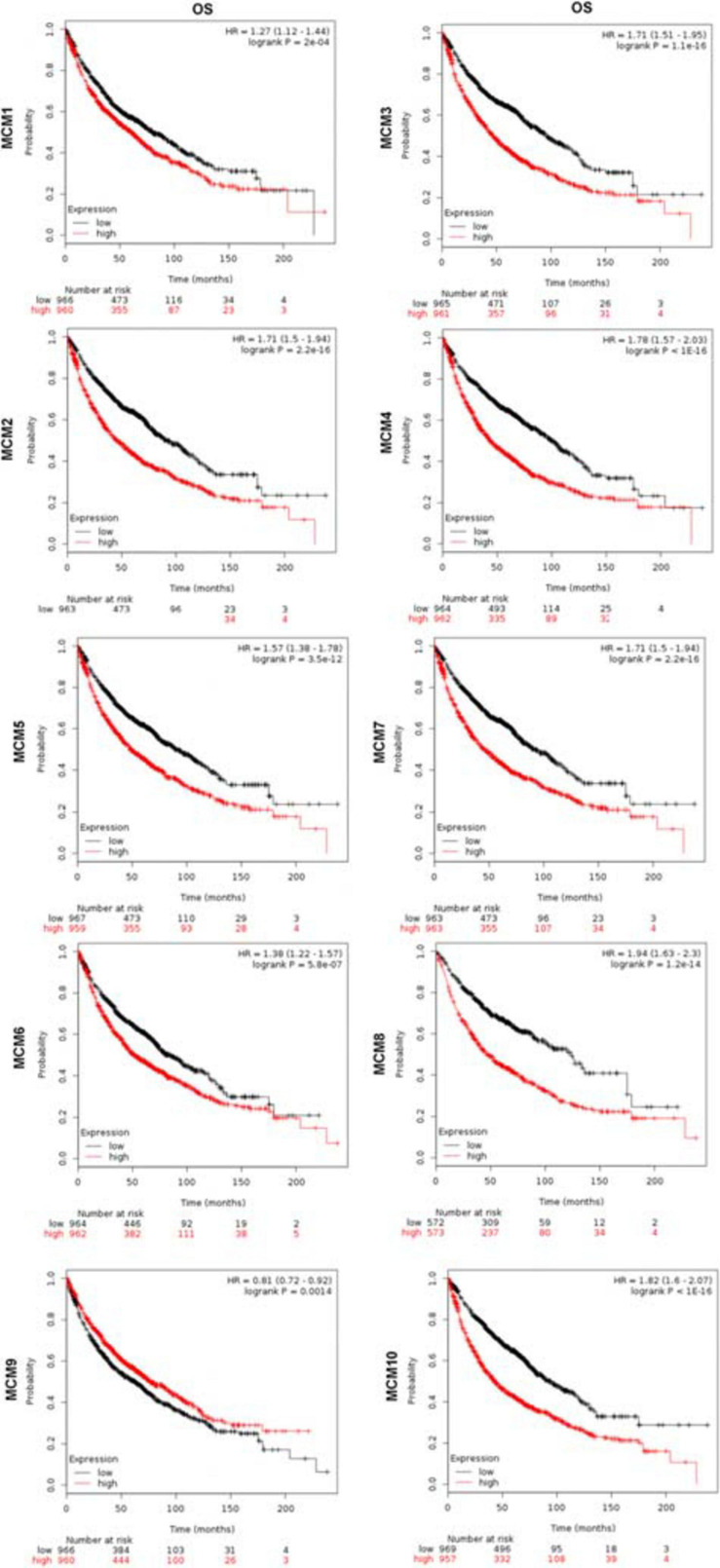
The prognostic value of mRNA level of MCMs in lung cancer patients by Kaplan–Meier plotter database.

Furthermore, we evaluated the prognostic value of MCMs in survival of NSCLC patients using the Kaplan–Meier Plotter tools (2015 version). The Kaplan–Meier curve and log-rank test analysis results showed that increased MCM1/2/3/4/5/6/7/8/10 mRNA levels and decreased MCM9 mRNA level were significantly associated with the OS of NSCLC patients. (Figure 4).

### The Mutation Status and Expression Correlations Among MCMs in NSCLC

**FIGURE 5:**
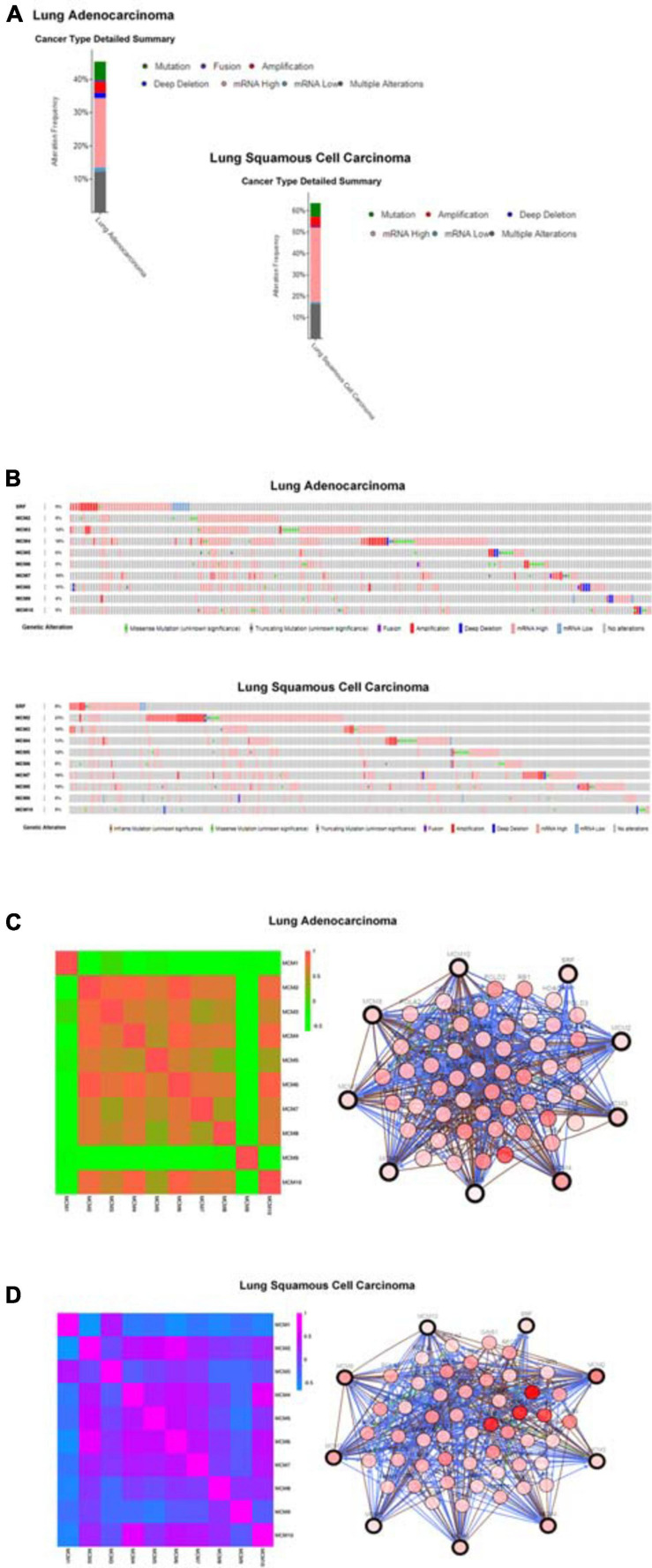
MCMs mutation analysis and correlations between each other or with other genes in LUAD and LUSC by cBioPortal database. **(A)** The alteration frequencies of MCM across NSCLC studies. The red bars indicate gene amplification, blue bars are deep deletions, green bars are non-synonymous mutations, pink bars are mRNA high expression, cyan bars are mRNA low expression and gray bars indicate multiple alterations. **(B)** Genetic alterations. Red represents amplification, blue represents deep deletion and pink represents mRNA up-regulation. **(C)** Network between MCMs and the 50 most frequently altered neighbor genes in LUAD samples. **(D)** Network between MCMs and the 50 most frequently altered neighbor genes in LUSC samples.

To explore the potential expression pattern of MCMs in NSCLC, we set out to analyzed the mutation status of each MCM and expression correlations among them by using the cBioPortal online tool. Queried MCMs were mutated in 228 out of 503 samples from patients with LUAD (45%), and 296 out of 466 samples from patients with LUSC (64%) (Figure 5A). In LUAD samples, the mutation rate of MCM4 was the highest, at a percentage of 18%, compared with other MCM proteins, while in LUSC samples, the highest mutation rate was found in MCM2, accounting for 23% (Figure 5B). The high frequency of mutation types in LUAD and LUSC mainly included amplification, missense mutation (unknown significance), mRNA high and deep deletion. Furthermore, to have a better understanding of the correlations among MCMs, we analyzed their mRNA levels in LUSC (TCGA, PanCancer Atlas) and LUAD (TCGA, PanCancer Atlas) *via* cBioPortal online tool. The results revealed significant positive correlations among MCM2/3/4/5/6/7/8/10 in LUAD patients. Specifically, expression of MCM2 with MCM3, MCM4, and MCM6; MCM3 with MCM2; MCM4 with MCM2 and MCM6; MCM6 with MCM2, MCM4, and MCM10; MCM10 with MCM6 showed a high level of correlation with each other and had a score of more than 0.7 (Figure 5C). Meanwhile, in patients with LUSC, we also found a significant associations of MCMs with each other. Expression of MCM1 with MCM3; MCM2 with MCM4, MCM5, MCM6, and MCM7; MCM3 with MCM1, MCM6, and MCM7; MCM4 with MCM2, MCM5, MCM6, MCM7, and MCM10; MCM5 with MCM2, MCM4, and MCM6; MCM6 with MCM2, MCM3, MCM4, MCM5, MCM7, and MCM10; MCM7 with MCM2, MCM3, MCM4, MCM6, and MCM10; and MCM10 with MCM4, MCM6, and MCM7 were highly correlated (Figure 5D). Next, we constructed a network between MCMs and the 50 most frequently altered neighbor genes in LUSC and LUAD samples, respectively. The results indicated that the DNA replication-related genes, including RAD1 and PRKDC, were closely associated with MCM alterations in LUAD patients (Figure 5C). Meanwhile, in patients with LUSC, the MCM alterations were closely associated with cell cycle- and DNA replication-related genes, such as RCF4, ATR, RAD1, and TOPBP1 (Figure 5D).

### Experimental Validation of the Increased Expression Levels of MCM2 and MCM4 in NSCLC Tissues

**FIGURE 6:**
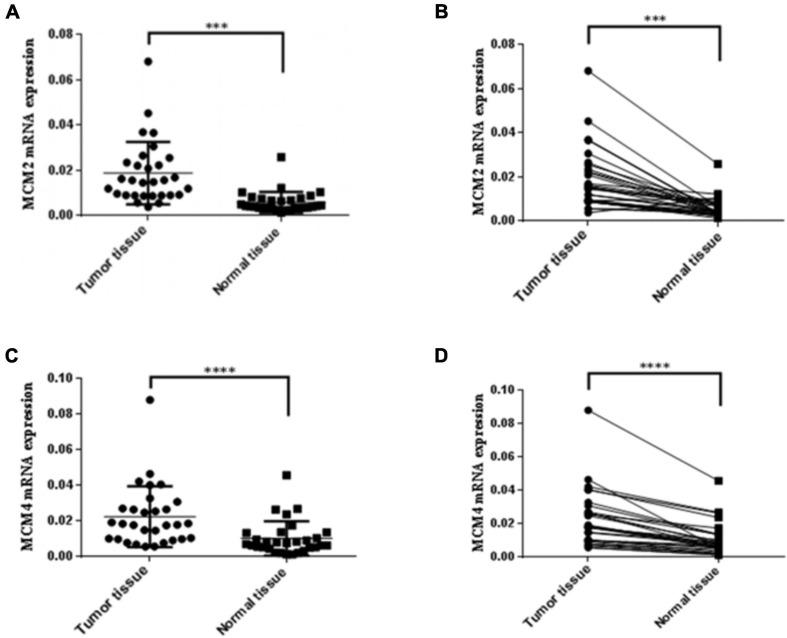
Expression of MCM2 and MCM4 were up-regulated in NSCLC tissues. **(A,B)** Comparison of the mRNA levels of MCM2 in NSCLC tissues and paired-adjacent normal lung tissues. **(C,D)** Comparison of the mRNA levels of MCM4 in NSCLC tissues and paired-adjacent normal lung tissues. *N* = 28, ^∗∗∗^*p* < 0.001, and ^*⁣*⁣**^p < 0.0001 based on the Student’s *t*-test.

Given that MCM2 and MCM4 not only constantly showed association with the clinical stages and OS, but also exhibited the highest mutation rate in LUAD and LUSC patients, respectively, we then set out to validate the overexpression of MCM2 and MCM4 in NSCLC tissues. The mRNA levels of MCM2 and MCM4 were determined by using NSCLC tissues and the paired-adjacent normal lung tissues. The qRT-PCR result showed that mRNA levels MCM2 and MCM4 were significantly increased in NSCLC tissues, compared to that in paired normal lung tissues (Figures 6A–D).

### The Potential Biological Functions MCM2 and MCM4 in NSCLC

**FIGURE 7:**
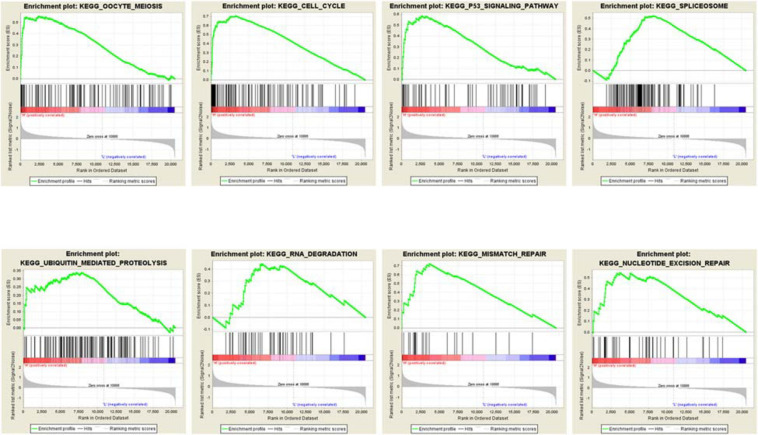
The GSEA analysis result of MCM2/4.

To further explore the potential biological functions MCM2 and MCM4 in LUAD and LUSC, we analyzed the correlation between MCM2/MCM4 levels and the involving biological processes by GSEA based on TCGA datasets. We spilt the samples into top 50 and bottom 50 of MCM2/4 levels (high- and low-level groups), and selected top 10 significant pathways in each group for further analysis (Table 2). Notably, eight out of 10 pathways were overlapped between MCM2 and MCM4 high-level groups, such as cell cycle, p53 signaling pathway, ubiquitin mediated proteolysis, mismatch repair and nucleotide excision repair (Figure 7). This result suggested that MCM2 and MCM4 are implicated in cancer progression through these pathways, and their overexpression correlates with poor prognosis for NSCLC patients.

**TABLE 2:** Top 10 of pathway enriched by GSEA analysis in MCM2 and MCM4.

	**Name of pathway**	**NES**	**NOM *p*-val**	**FDR *q*-val**
MCM2	Oocyte meiosis	1.88	0	0.266
	Cell cycle	1.87	0	0.151
	P53 signaling pathway	1.84	0	0.124
	Lysine degradation	1.82	0.004	0.113
	Spliceosome	1.82	0.013	0.091
	Ubiquitin mediated proteolysis	1.73	0.031	0.157
	RNA degradation	1.7	0.039	0.163
	Mismatch repair	1.7	0.002	0.148
	Base excision repair	1.68	0.025	0.152
	Nucleotide excision repair	1.67	0.027	0.143
MCM4	Oocyte meiosis	1.89	0.002	0.169
	Cell cycle	1.89	0	0.087
	Spliceosome	1.85	0.008	0.074
	Ubiquitin mediated proteolysis	1.85	0.004	0.059
	P53 signaling pathway	1.79	0.006	0.09
	RNA degradation	1.78	0.038	0.081
	Basal transcription factors	1.75	0.006	0.088
	Nucleotide excision repair	1.71	0.022	0.108
	Mismatch repair	1.7	0.004	0.111
	DNA replication	1.69	0.002	0.103

## Discussion

Minichromosome maintenance proteins represent potentially more accurate proliferative biomarkers to determine the proliferative fraction within a tumor, compared to the conventional proliferation index such as Ki-67, as MCMs are downregulated later when the cells adopt a terminally differentiated phenotype. MCM2-7 complex proteins can be detected throughout the cycle except in quiescence (G0), thus they can be used as general markers of tumor growth fractions (Maros et al., 2019). Furthermore, as MCMs play an essential role in DNA replication, they are closely related to the initiation and development of tumors (Deegan and Diffley, 2016; Fei and Xu, 2018; Meagher et al., 2019; Wang H.Y. et al., 2019). Evidence from tumor genome sequencing shows that dys-regulation of Rb/E2F and G1/S phase can aggravate oncogenic replication stress, resulting in genomic instability caused by DNA double-strand break (DSB), and subsequent loss of key regulators such as the p53 tumor suppressor, and eventually lead to tumorigenesis. For instance, MCMs are the targets of E2F, thus E2F can promote tumorigenesis through MCMs by enhancing cell proliferation (Ha et al., 2004). Moreover, MCMs can identify not only cycling cells but also non-cycling cells with proliferative potential.

The research on MCM1 and MCM9 has not been previously reported. Results from this study show that there was no significant difference in the mRNA levels of these MCMs between lung cancer and normal lung tissues, and neither of them correlated with tumor stage. In terms of survival rate, MCM1 was found to negatively associate with OS, while the high level of MCM9 was related to better OS, which was not consistent the role of other MCMs on the prognosis of lung cancer. MCM2 is a dominant MCM protein, and its upregulation was significant associated with advanced clinical stage, large tumor size, more lymph node and distant metastasis (Wu et al., 2018). Also, overexpression of MCM2 was an independent unfavorable prognostic factor for LUSC patients (Wu et al., 2018). Zhang Z.H. et al. (2015) found that MCM2 could promote cell proliferation by regulating HMCMGA1 phosphorylation, which was previously reported to be related to the malignant status and poor prognosis of NSCLC. MCM2 has also been reported as a potential therapeutic target of lovastatin for NSCLC (Zhang X. et al., 2015). MCM4 is one of the six MCM proteins composing the prereplicative complex that binds to replication origins in the G1 phase of the cell cycle, and it was essential for the initiation of DNA replication (Kikuchi et al., 2011). MCM4 was essential for the growth of NSCLC, thus it was considered as a potential therapeutic target for NSCLC patients. In this study, our bioinformatics analysis results based on public datasets showed that MCM2 and MCM4 were overexpression of in NSCLC, which positively correlated with poor prognosis in NSCLC patients. Moreover, we validated the overexpression of MCM2 and MCM4 in NSCLC by using the fresh samples from 30 patients who had undergone tumor removal surgery in our hospital. Intriguingly, the expression levels of MCM2 and MCM4 were significantly higher in NSCLC tissues than those in normal lung tissues, although the cohort size was relatively small.

MCM3 has been found to overexpress in various of cancers, and the specific high-expression in cancerous cells rendered it an ideal biomarker for detecting malignant cells (Ha et al., 2004). Consistently, our result showed that MCM3 was highly expressed in lung cancer, especially in LUSC, and high MCM3 level was significantly correlated with poor OS in all subtypes of lung cancer patients. MCM5 was also highly expressed in many tumors, such as thyroid cancer cells (Mio et al., 2016^)^ and breast cancer (Eissa et al., 2015), but its expression status and prognostic value in lung cancer have yet to be evaluated. Our results showed that the expression level of MCM5 in lung cancer tissues was significantly higher than that in normal tissues, and high MCM5 expression is significantly correlated with poor OS. MCM6 has been reported to associate with the prognosis of NSCLC (Kadara et al., 2009). Knock-out of TMCMPRSS4 could lead to down-regulation of MCM6 in NSCLC, which in turn leads to down-regulated proliferation and migration of cancer cells (Exposito et al., 2019). Here, we found a relatively high expression of MCM6 in LUAD and LUSC compared to normal lung tissue, and confirmed that high expression of MCM6 has a negative impact on the prognosis in NSCLC patients.

As a key member of the MCM family, MCM7 plays an important role in maintaining the stability of the initial process during DNA replication (Chuang et al., 2012). It ensures DNA replication to occur only once within a cycle. In view of this, overexpression of MCM7 may result in an increased cellular proliferative rate. A previous study showed that high MCM7 expression was associated with male gender, non-adenocarcinoma histology and poor tumor differentiation in NSCLC patients (Kikuchi et al., 2011). In NSCLC cells, YAP/TAZ could induce the transcription of the MCM7 gene and hosted miRs (miR-25, 93, and 106b cluster), thereby promoting cell proliferation through the post-transcriptional inhibition of the p21 (Wang H.Y. et al., 2019). Also, RACK1 could regulate the cell growth and cell cycle progression in NSCLC by mediating MCM7 phosphorylation through an MCM7/RACK1/Akt signaling complex (Fei et al., 2017). In addition, MCM7 was reported to associate with mRNA transcription and DNA damage (Fujii et al., 2018). MCM8 has only been reported to implicate in female reproductive function, and no evidence linked it to lung cancer so far, whereas MCM10 has previously been found to implicate in breast cancer and uveal melanoma (Johnson et al., 2003; Chen et al., 2014; Maros et al., 2019). In this study, we found that MCM7 was highly expressed in LUAD, while MCM8/10 were overexpressed in large cell lung cancer and LUSC, all of which negatively associated with patient’s OS. Taken together, aiming to systematically investigate the distinct expression patterns and prognostic values of MCMs in lung cancer, we analyzed their transcriptional levels, mutations and association with the key clinico-pathological parameters of lung cancer patients by using a bioinformatics approach, and the potential biological roles of MCMs in lung cancer were also explored, which provided an insight into the clinical application of MCMs as potential prognostic and therapeutic indicators.

## Conclusion

In this study, we set out to analyze the expression levels, mutation patterns, and prognostic value of MCMs in NSCLC patients, and further explored the potential roles of MCMs in lung cancer, which provided a holistic view of the heterogeneity and complexity of the biological characteristics of MCMs. The results from this study support a view that MCM2-10 may serve as prognostic biomarkers for NSCLC. Specifically, overexpressed MCM2 and MCM4 may play important roles in NSCLC tumorigenesis, and they could serve as potential indicators to identify high-risk subgroups of NSCLC patients.

## Data Availability Statement

The original contributions presented in the study are included in the article/Supplementary Material, further inquiries can be directed to the corresponding author/s.

## Author Contributions

LL, WC, and YC designed and supervised the study and finalized the manuscript. CH, CL, BP, and SF contributed to the study. All authors contributed to the article and approved the submitted version.

## Conflict of Interest

The authors declare that the research was conducted in the absence of any commercial or financial relationships that could be construed as a potential conflict of interest.

## Footnotes

1 www.oncomine.org

2 www.kmplot.com

3 http://www.cbioportal.org/index.do?session_id=5a37ba8e498eb8b3d56242fb

4 https://tcga-data.nci.nih.gov/tcga/

5 http://gepia.cancer-pku.cn/

## Abbreviations

LC: Lung cancer
OS: overall survival
NSCLC: non-small cell lung cancer
SCLC: small cell lung cancer
LUAD: lung adenocarcinoma
LUSC: squamous cell lung carcinoma
MCMs: Minichromosome maintenance proteins
GEPIA: Gene Expression Profiling Interactive Analysis
FP: progression-free survival
PPS: post progression survival
GO: Gene Ontology
KEGG: Kyoto Encyclopedia of Genes
DAVID: Database for Annotation Visualization and Integrated Discovery
BP: biological processes
CC: cellular components
MF: molecular functions
GSEA: Gene Set Enrichment Analysis
HR: homologous recombination
PTMs: posttranslational modification
SRF: Serum response factor.
