# Supplementary material for: Potential Prospective Biomarkers for Non-small Cell Lung Cancer: Mini-Chromosome Maintenance Proteins
Source: Front Genet. 2021 Apr 14;12:587017. doi: 10.3389/fgene.2021.587017 (PMC8079985; doi:10.3389/fgene.2021.587017)
Supplement: Supplementary file 1 [file Table_1.DOCX]

**Supplementary Table 1.** **Primer sequences of mRNA structural components used in qRT-PCR**

| **Components** | **Position** | **Sequence** |
| --- | --- | --- |
| MCM2 | Forward | ATGGCGGAATCATCGGAATCC |
|  | Reverse | GGTGAGGGCATCAGTACGC |
| MCM4 | Forward | TGAACCTCTATACATGCAACGAC |
|  | Reverse | CAGGGTAACGGTCAAAGAAGATT |
| GAPDH | Forward | TGCACCACCAACTGCTTAGC |
|  | Reverse | GGCATGGACTGTGGTCATGAG |
